# Genetic progression in gastrointestinal stromal tumors: mechanisms and molecular interventions

**DOI:** 10.18632/oncotarget.16014

**Published:** 2017-03-08

**Authors:** Ke Li, Haibo Cheng, Zhang Li, Yuzhi Pang, Xiaona Jia, Feifei Xie, Guohong Hu, Qingping Cai, Yuexiang Wang

**Affiliations:** ^1^ SIBS (Institute of Health Sciences), Changzheng Hospital Joint Center for Translational Medicine, Institute of Health Sciences, Shanghai Changzheng Hospital, Institutes for Translational Medicine (CAS-SMMU), University of Chinese Academy of Sciences, Shanghai, China; ^2^ Key Laboratory of Stem Cell Biology, Institute of Health Sciences, Shanghai Institutes for Biological Sciences, Chinese Academy of Sciences and Shanghai Jiao Tong University School of Medicine, Shanghai, China; ^3^ Collaborative Innovation Center of Systems Biomedicine, Shanghai Jiao Tong University School of Medicine, Shanghai, China; ^4^ The First Clinical Medical College, Nanjing University of Chinese Medicine, Nanjing, China; ^5^ Key Laboratory of SATCM for Empirical Formulae Evaluation and Achievements Transformation, Nanjing, China; ^6^ Collaborative Innovation Center of Jiangsu Province Chinese Medicine in Cancer Prevention and Treatment, Nanjing, China; ^7^ Department of Gastro-intestinal Surgery, Changzheng Hospital, Second Military Medical University, Shanghai, China

**Keywords:** gastrointestinal stromal tumors, dystrophin, small molecules, targeted therapy

## Abstract

Gastrointestinal stromal tumors (GISTs) are the most common sarcomas in humans. Constitutively activating mutations in the KIT or PDGFRA receptor tyrosine kinases are the initiating oncogenic events. Most metastatic GISTs respond dramatically to therapies with KIT/PDGFRA inhibitors. Asymptomatic and mitotically-inactive KIT/PDGFRA-mutant “microGISTs” are found in one third of adults, but most of these small tumors never progress to malignancy, underscoring that a progression of oncogenic mutations is required. Recent studies have identified key genomic abnormalities in GIST progression. Novel insights into the genetic progression of GISTs are shedding new light on therapeutic innovations.

## INTRODUCTION

Gastrointestinal stromal tumors (GISTs) are common neoplasms that originate in the mesenchymal tissue of the gastrointestinal tract, and so-called micro GISTs are found in ∼20-30% of adults [1–3]. Malignant, metastatic GISTs serve as a clinical paradigm for inhibiting activating driver mutations to confer a major clinical benefit to patients and a rational clinical model to evaluate new targeted therapies and identify molecular mechanisms of drug response and resistance [4]. However, the genetic events responsible for the clinical progression of this tumor type are poorly understood. GISTs are generally considered to emanate from the interstitial cells of Cajal (ICC), which are pacemaker cells regulating gut motility. ICCs originate from the same precursor cells as smooth muscle tissue, common intestinal mesenchymal precursor cells [5]. GISTs are most frequently found in the stomach, small intestine, colon, esophagus, and rectum [1, 6, 7]. Clinical GIST is a relatively rare cancer; the average annual incidence ranges from 11 to 19.6 per million individuals [4].

In the last few decades, several genetic events in the initiation and progression of GIST have been reported. Specifically, a large portion of the GISTs harbor oncogenic mutations in KIT or platelet-derived growth factor receptor-α (PDGFRA) [8–12], and some GISTs without KIT or PDGFRA mutations are called wild-type GISTs. This review provides an overview of inspiring developments in the oncology, pathology and pharmacology of GISTs. The focus is placed on the driver mutations and chromosomal alterations involved in GIST initiation and progression as well as the relationship between resistance to targeted therapeutics and secondary mutations.

## KIT/PDGFRA GAIN-OF-FUNCTION MUTATIONS

### *KIT* mutations

KIT, also known as CD117, is a member of the type III receptor tyrosine kinase family [8]. The family consists of PDGFRA, PDGFRB, FLT3 (Fms-like tyrosine kinase 3) and CSF1R (macrophage colony-stimulating-factor receptor) [13]. KIT protein is expressed in several cell types, including mast cells, melanocytes, hematopoietic progenitor cells, germ cells, and ICCs. Normally, KIT does not have kinase activity until it binds stem cell factor (SCF) and homodimerizes [14].

**Figure 1:**
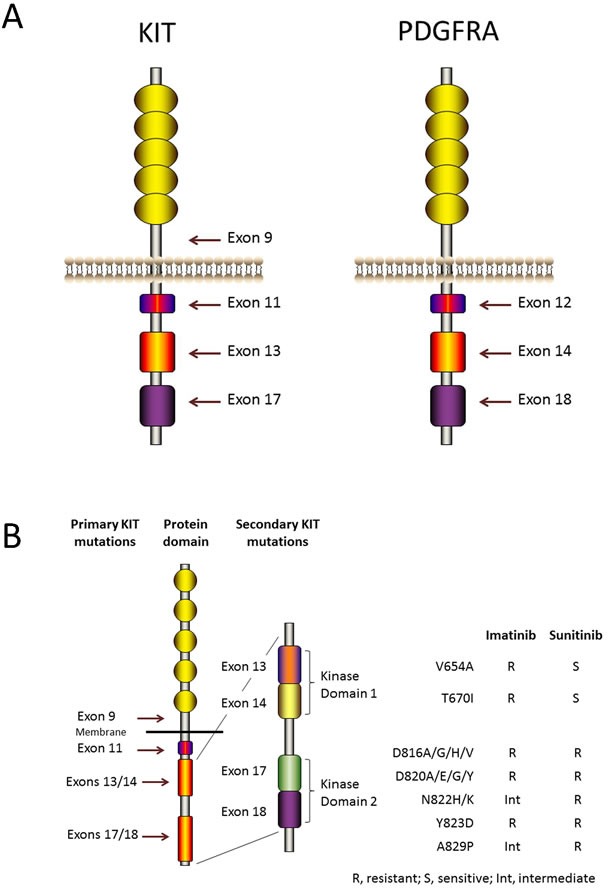
KIT and PDGFRA structure and mutations **A.** KIT and PDGFRA are type III receptor tyrosine kinases and have the same topology: an extracellular ligand-binding domain that consists of five immunoglobulin-like repeats, a transmembrane sequence, a juxtamembrane domain and a cytoplasmic kinase domain. Mutations in the juxtamembrane domain, which is encoded by exon 11 of *KIT* or by exon 12 of *PDGFRA*, allow receptor dimerization without ligands and modulate the kinase activation loop from swinging to activating. Mutations in the activation loop, which is encoded by exon 17 of *KIT* or by exon 18 of *PDGFRA*, and mutations in the ATP-binding region, which is encoded by exon 13 of *KIT* or by exon 14 of *PDGFRA*, all stabilize the active conformation of KIT or PDGFRA. Mutations in the extracellular domain of KIT (encoded by exon 9) favor receptor dimerization. **B.** Secondary KIT mutations and drug sensitivities. Secondary mutations cluster in two regions of the KIT oncoprotein: the tyrosine kinase domain (TKD) 1 (encoded by exons 13 and 14) and the TKD 2 (encoded by exons 17 and 18).

Moreover, 78.5% GISTs contain *KIT* mutations (Figure 1A), and these alterations contribute to the constitutive activation of the KIT oncoprotein. Therefore, mutant KIT is a crucial diagnostic marker and clinical therapeutic target for the treatment of GISTs [15].

Oncogenic KIT mutants result in spontaneous receptor homodimerization and kinase activation without SCF [16]. The most frequent mutations occur in exon 11 of *KIT*, which encodes the juxtamembrane domain. Specifically, 85% of *KIT* mutations are caused by mutations in exon 11, and these mutations change the juxtamenbrane structure and modulate the KIT protein activation loop from swinging to activating [17, 18]. In addition to exon 11 mutations, approximately one tenth of *KIT* mutations occur in exon 9, which encodes an extracellular domain. The conformations of these mutant KITs are similar to that of the extracellular domain bound with KIT ligand [19]. Besides, other uncommon mutations, such as mutations in the KIT activation loop and mutations in the ATP-binding pocket, encoded by exon 17 and exon 13, respectively, occur, too [20, 21]. These mutations all stabilize the active structure of KIT.

**Figure 2:**
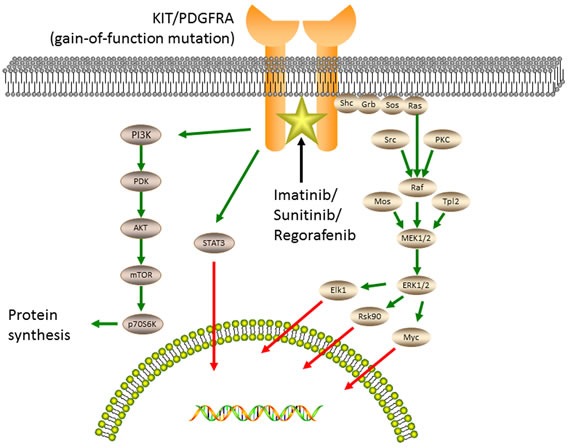
Oncogenic tyrosine kinase signaling and accessory pathways responsible for the pathogenesis of GISTs The kinase activation of KIT or PDGFRA stimulates downstream signaling pathways, including the MAPK pathway, the PI3K-AKT pathway and the STAT3 pathway. Imatinib, sunitinib and regorafenib inhibit several kinases, including KIT and PDGFRA, by binding to the ATP-pocket and yield durable responses.

The KIT oncoprotein activation stimulates downstream signaling pathways (Figure 2), including the PI3K-AKT pathway, the MAPK pathway, and the STAT3 (signal transducer and activator of transcription 3) pathway [4, 22].

### *PDGFRA* mutations

PDGFRA shares similar homologue with KIT. Approximately 5-8% GISTs contain a mutation in *PDGFRA* gene (Figure 1A). Immunoblots of tumor samples have shown that a minority of GISTs without *KIT* mutations exhibit high levels of PDGFRA phosphorylation [11]. Specifically, activated PDGFRA is detected in GISTs that harbor mutations in exons 12, 14 and 18, which encode the PDGFRA juxtamembrane domain, and the kinase domains, respectively. What's more, mutant PDGFRA exhibits activated kinase activity without their ligand PDGFA. It shares the same downstream signaling pathways with those in *KIT*-mutant GISTs. Furthermore, heat shock protein 90 (HSP90) stabilizes mutant PDGFRA [23, 24].

In addition to these similarities, several pathological features distinguish PDGFRA-mutant GISTs from KIT-mutant GISTs, such as different whole genome expression profiles, a significantly higher morbidity in the stomach, variable KIT expression and a lower possibility for malignancy [9, 25, 26]. Nevertheless, the mechanisms for these distinctions remain unclear.

### *KIT/PDGFRA* inhibitors

Because of these mutations in KIT and PDGFRA, tyrosine kinase inhibitor (TKI) therapy has been developed to treat GISTs. TKIs have revolutionized the treatment of GISTs in the last 15 years [27–30], and three TKIs have been approved to treat GIST (Table 1). Specifically, imatinib was approved as the standard first-line drug. It inhibits several kinases, including KIT and PDGFRA, by binding to the ATP-binding domain and induces dramatic disease control in about 85% of GISTs. In addition, it has shown excellent initial clinical responses in approximately 80% of GIST patients, with only 10-15% of patients exhibiting primary resistance [28, 29, 31]. More specifically, imatinib produces higher partial response rates in GISTs with the exon 11 *KIT* mutation than in GISTs with the exon 9 mutation or patients without a *KIT* or *PDGFRA* mutation. Conversely, patients with tumors harboring *PDGFRA* mutations (D842V) are strongly resistant to imatinib [32–34]. Therefore, mutational tests for *KIT* and *PDGFRA* should be considered for metastatic or advanced GIST treatment planning. In 2006, the median survival for patients being treated with imatinib was 4.8 years. Although imatinib shows remarkable initial success in the treatment of GISTs, its long-term efficacy is not as dramatic because many secondary mutations in *KIT* cause the tumors to become resistant [35]. Similar to imatinib, sunitinib inhibits KIT and PDGFRA activities by binding to the ATP-binding domain. However, the binding features of sunitinib differ from those of imatinib. Sunitinib can inhibit the vascular endothelial growth factor receptor (VEGFR) and the rearranged during transfect (RET) kinase as well. Thus, sunitinib was approved to combat imatinib resistance in 2006. In a clinical study, 312 patients were given with sunitinib for four weeks at a daily dose of 50 mg, and followed by a two-week break. The result showed that in the sunitinib and placebo arms, the median progression-free survival times were 6.3 and 1.5 months with a hazard ratio (HR) of 0.33. Compared with the placebo arm, overall survival was superior in the sunitinib group [36]. However, sunitinib likely caused significant side effects, including skin pigmentation disorders, fatigue and so on. Thus, the benefits of sunitinib therapy are conserved than those of first-line imatinib standard treatment [36]. However, the inherent heterogeneity of resistant mutations in GIST tumors precludes the universal efficacy of sunitinib [21]. Regorafenib is an oral kinase inhibitor that binds several kinases functioned in tumorigenesis, including KIT and PDGFRA [37–40]. The most frequent side effects were hand-foot skin reactions and hypertension.

**Table 1:** FDA approved TKIs for the treatment GISTs

Drug	Key molecular targets	Manufacturer	Setting tested	Common dose	Frequent adverse effects	Chemical structure
Imatinib (Gleevec®)	KIT, PDGFRA, ABL, FLT3, CSF1R, SD	Novartis	First line	400mg	Nausea, diarrhea, headaches, leg aches/cramps, fluid retention, visual disturbances	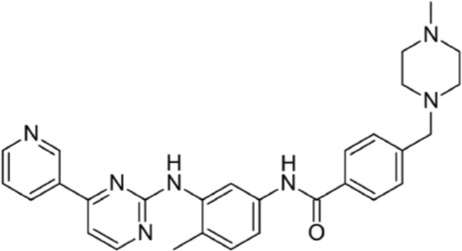
Sunitinib (Sutent®)	KIT, PDGFRA, VEGFR, RET	Pfizer	Second line	37.5mg	Anemia, neutropenia, fatigue, diarrhea, skin discoloration, nausea, anorexia	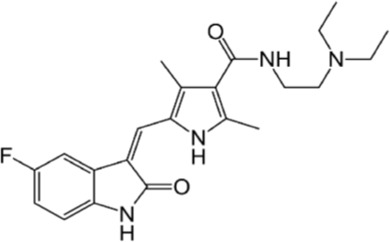
Regorafenib (Stivarga®)	KIT, PDGFRA, RET, RAF1, BRAF, VEGFR1-3, TIE2, FGFR	Bayer	Third line	160mg	Hand-foot skin reaction, hypertension, diarrhea	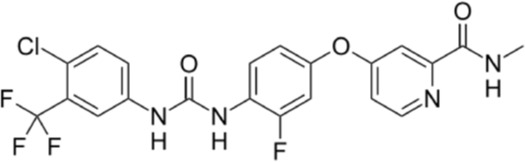

## CDKN2A/P53 DYSREGULATIONS

*CDKN2A* (cyclin-dependent kinase inhibitor 2A) is a tumor suppressor gene encoding the p16 and p14. p16 suppresses cyclin dependent kinases 4 and 6 (CDK4 and CDK6) and thus activates the retinoblastoma (Rb) family members, which inhibit cell cycle from the G1 to the S phase. p14 activates the p53 tumor suppressor. Furthermore, several studies indicated that CDKN2A significantly impacts the pathogenesis of GIST [41, 42], and most advanced GISTs harbor *CDKN2A* inactivation due to chromosome 9p21 deletion, which may be biallelic or combined with other mutations or promoter methylation [43, 44]. In 2015, the Food and Drug Administration (FDA) approved ibrance to treat (in combination with letrazole) breast cancer patients with human epidermal growth factor receptor 2 (HER2)(-) and estrogen receptor (ER) (+). Since ibrance is a selective inhibitor of CDK4 and CDK6, it may be efficacious to treat GISTs. Adverse reactions to ibrance include neutropenia, infections and pulmonary embolism.

p53 protein, encoded by the TP53 gene in humans, also acts as a tumor suppressor. It participates in many biological processes, including cell cycle, programmed cell death and DNA repair. Mutations in the *TP53* gene frequently occur in neoplasms, including GISTs, and these mutations result in p53 protein inactivation. Most mutations in *TP53* are centered in exons 4 to 8, which are also the most highly conserved exons in vertebrates. These mutations alter the central DNA-binding core domain. The TP53 protein binds to DNA and has the following three main functions: activating DNA repair mechanisms, holding the cell cycle at the G1/S regulation point to allow DNA repair and consequently arrest cell growth, and initiating apoptosis. These functions lead to cell stability, but when *TP53* is mutated, the cell is allowed to grow and divide uncontrollably, leading to cancer. Another gene, *MDM2*, encodes a protein that negatively controls TP53 by degrading it, and MDM2 is induced by TP53 in a negative feedback loop. However, MDM2 is not induced by mutated TP53, and mutated TP53 consequently accumulates at very high concentrations. Some studies showed that impaired p53 expression is common in advanced GISTs, and p53 has a strong effect on the progression-free survival. Moreover, the accumulation of p53 protein correlated significantly with the mitotic rate and the risk of malignancy. Mutations in *TP53* or reduced p53 expression are associated with an unfavorable prognosis [45, 46].

## DYSTROPHIN INACTIVATION

### Dystrophin tumor suppressor in GIST

**Figure 3:**
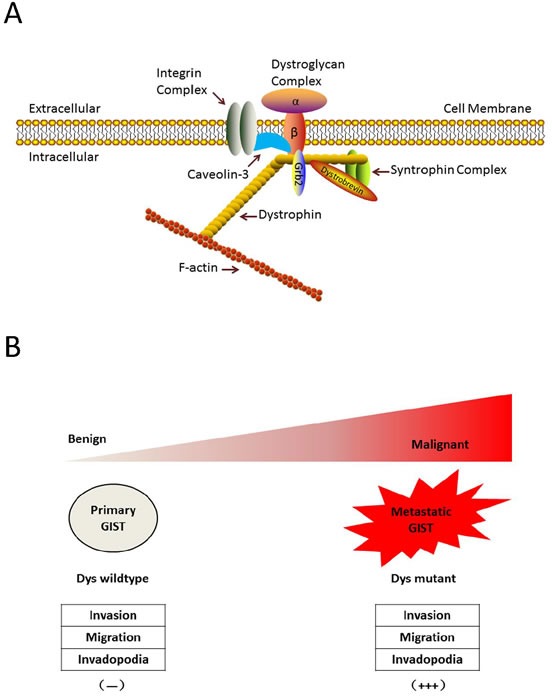
Dystrophin is a novel tumor suppressor that regulates GIST invasion, migration, anchorage-independent growth and invadopodia formation **A.** Dystrophin structure. Dystrophin is a rod-shaped structure protein, forming a protein complex providing a link between the cytoskeleton of a muscle fiber and the surrounding extracellular matrix. **B.** Myogenic dystrophin 427kDa isoform was expressed strongly (and had no demonstrable genomic mutations) in low-risk primary GISTs, whereas dystrophin expression was undetectable in most metastatic GISTs, most of which had inactivating intragenic *DMD* mutations. Dystrophin inactivation increases cell invasion, migration, anchorage-independent growthand invadopodia formation.

Dystrophin is a rod-shaped structure protein, forming a protein complex providing a link between the cytoskeleton of a muscle fiber and the surrounding extracellular matrix (Figure 3A). In cancer, dystrophin suppresses many cell behaviors, such as cell migration, invasion, anchorage independence, in sarcomas with myogenic features. A recent study showed that the somatic *DMD* deletion is a common mechanism by which myogenic tumors progress to advanced sarcomas (Figure 3B). *DMD* is one of the largest human genes and consists of 79 coding exons that span 2.2 Mb of the genome, with multiple isoforms. *DMD* deletions target 427-kDa myogenic isoform, while the expression of a 71-kDa isoform, which is essential for cell viability, is preserved. Dystrophins are expressed highly in the ICC cells and low-risk GISTs, whereas dystrophin inactivation was identified in 96% of metastatic GISTs [47].

*DMD* is an X-linked tumor suppressor gene, but the deletion rates do not differ between males and females. Specifically, copy number profiles demonstrated that the inactive X chromosome was intact in female GISTs with heterozygous *DMD* mutations. Both heterozygous and homozygous *DMD* mutations in females resulted in entire *DMD* knockout. We demonstrated that dystrophin dysregulation can be used as a prognostic factor in GISTs and as a novel target of attack for GIST therapy. This is a significant finding that impacts the direction of future research studies. The implication is that by detecting key genetic defects such as the lack of dystrophin in GIST patients, it may eventually become possible to predict those who will develop metastatic diseases. These studies determine whether loss of dystrophin expression identifies a high-risk group of patients, with higher frequency of subsequent metastases, and lower recurrence-free and overall survival, compared to patients whose primary tumors retain dystrophin expression. *DMD* deletions are enriched in myogenic cancers, and *DMD* inactivation selectively increases metastatic potential in GISTs. What's more, the knockdown of the *Dp71* isoform decreased cell viabilities, showing that 71-kDa isoform is required for cancer cell growth. Moreover, dystrophin interacts with dystrophin glycoprotein complex. The evidence of dystrophin tumor suppressor suggests that other components in dystrophin complex are likely to play key roles in GIST tumorgenesis [48, 49].

### Targeting dystrophin deficiency

Some studies have attempted to correct the dystrophin gene reading frame by modulating pre-mRNA splicing with antisense oligonucleotides (AOs)-mediated exon skipping, which has shown success both *in vitro* and *in vivo*. Genetic testing can detect the precise character and *DMD* mutation site. *DMD* mutations in muscular dystrophy cluster in hotspot regions, which are mainly located on exons 45-53 and exons 2-20. Most patients with DMD would benefit from the creation of generally needed AOs. Specifically, up to 50% of these patients could benefit from AO cocktails to produce the skipping of the exons 45-53 [50–52].

### Recent studies demonstrate that the functions of utrophin and dystrophin are equivalent in muscle

Dystrophin-deficient *mdx* mouse can be cured by utrophin therapy. Utrophin mRNA levels can be increased through its promoter manipulation. Similarly, SMT C1100 is invented to increase and maintain utrophin transcription. It induces increased utrophin mRNA and protein levels in human muscle cells and reduces dysrophin-deficient muscle pathology. Furthermore, it provides significant benefits to whole body strength and endurance. In theory, the development of small molecules (such as SMT C1100) can increase the level of utrophin to treat DMD. The most significant advantage of this approach is that it can treat all disease associated with DMD deficiency, irrespective of the dystrophin mutation [53].

Some studies show that the 70-kDa heat shock protein 1 (Hsp72) inducer and Wnt7a help to ameliorate muscular dystrophy. Intracellular Ca2+ is deregulated in dystrophic muscle fibers, which inducing inflammation. Inflammation contributes to dystrophic pathology via the pro-inflammatory cytokine TNF-α, which activates the NF-kB and JNK signaling pathways. Accordingly, dystrophin-deficient *mdx* mice treated with BGP-15, a Hsp72 activator, showed improved muscular dystrophy phenotype [54].

The non-canonical Wnt receptor Frizzled-7 is selectively expressed in satellite stem cells. Wnt7a protein induces the expansion of satellite stem cells, and its overexpression enhances muscle regeneration. In addition, Wnt7a activity results in muscle hypertrophy, and a preclinical study showed that Wnt7a can increase the specific force in dystrophic mice [55].

Dystrophin expression does not reduce the number of viable cells in GIST. More importantly, dystrophin deficiency could be detected in both TKI-sensitive and TKI-resistant GIST, suggesting the limited value of targeting dystrophin deficiency to overcome TKI resistance.

## 1P, 14Q, 22Q ALTERATIONS

Although gene mutations play a key role in GIST tumorigenesis, other molecular events are crucial in GIST progression [56], such as chromosomal alterations [57]. Among these chromosomal alterations, losses of chromosome arms 1p, 14q and 22q are the most commonly detected abnormities. According to research by Maria Debiec-Rychter et al., 11 tumors from 39 GIST samples harbored chromosome 1 aberrations. Specifically, one sample exhibited a loss of whole chromosome 1, one sample displayed a loss of 1q32-qter and the other seven tumors showed copy number losses in different bands of 1p [58, 59]. Furthermore, Julie Breiner's research indicated that a deletion of the long and/or short arms of chromosome 1 is related to the malignancy of GIST [60]. Chromosome 1 copy number abnormities look like as a secondary change that is crucial in GIST tumorgenesis, and chromosome arm 14q changes were detected in both primary and recurrent tumors. About 67% of GIST samples exhibit either total chromosome 14 loss or a partial 14q loss. According to the copy number and loss of heterozygosity (LOH) studies, tumor suppressor genes, that promote GIST initiation, may locate in two bands of the chromosome 14. Specifically, loss of 14q11.2 involving the *PARP2*, *APEX1* and *NDRG2* and loss of 14q32 involving the *SIVA*, and the inactivation of these genes may be important in the pathogenesis of GIST. A deletion of the long arm of chromosome 22 is found in about half GISTs, and this loss is invariably related to chromosome 1 aberrations. Moreover, losses of 22q are related to a poor prognosis. What's more, another tumor suppressor gene *NF2* is located in 22q12. Copy number losses of chromosomes 14 and 22 were identified in low-risk, intermediate-risk and high-risk GISTs, showing that these losses may occur early in GIST progression [61, 62].

## TKI RESISTANCE

### TKI resistance mechanisms

GISTs are resistant to traditional chemotherapeutic regimes and radiation treatments because of the dose-limiting effects on nearby vital organs. Based on studies with long-term follow up, adverse events, including recurrence, metastases, and death, occur in up to 85% of patients who have undergone surgical resection. Prior to the development of highly specific TKIs, no treatment options existed for patients with malignant, non-operable GISTs. Targeted TKIs work by competitively binding to the ATP binding domain of the tyrosine kinase active site in protein products of oncogenes, such as *KIT* [28], *ABL* [27, 63], *BCR-ABL* [63], and *PDGFRA* [32]. In the presence of the drug, ATP cannot bind and activate the protein, and the protein is consequently sustained in an inactive conformation. While in the inactive state, the proteins cannot dimerize, and downstream enzymatic activity is ultimately blocked, which halts cell growth.

The tyrosine kinase inhibitor imatinib was originally developed to treat CML, a previously fatal disease. However, the five-year survival rate of this disease was 95% in 2008, and imatinib has extended the life expectancy of these patients from 3-5 years to 30 years [63, 64]. After being FDA approved for the treatment of CML, imatinib was approved as the first-line drug for metastatic and recurrent GIST. However, the majority of patients will develop secondary resistance within two years [65, 66]. Most secondary resistance has been established to be caused by a secondary mutation in *KIT* or *PDGFRA*, and these secondary mutations are in *cis* with the primary mutation without exception [21, 67–71]. In a Phase 2 study, imatinib was used to treat advanced GISTs. The results showed that two-thirds of patients with imatinib refractory had a secondary *KIT* mutation [72]. These mutations especially frequently occurred in exon 11-mutated GISTs, while wild-type GISTs were not the case. The secondary mutations cluster in the following 2 regions of the KIT kinase domain that were targeted by imatinib: the ATP-binding pocket, which is encoded by exons13 and 14 whose mutations directly hinder drug binding, and the activation loop, which is encoded by exons 17and 18 whose mutations make KIT oncoprotein more stable in the active conformation and interfere with TKI interation [67, 73–76]. Several secondary mutations are relatively common, including V654A [77], a point mutation found in KIT exon 13, which is involved with the KIT ATP-binding pocket, and D820A or various point mutations involving N882, which are located in exon 17 in the activation loop of the protein (Figure 1B). Sometimes, the so-called “gatekeeper” mutation, T670I, is also seen in exon 14, which, like V654A, is involved with the KIT ATP-binding pocket [78]. The secondary mutations V654A and T670I are intrinsically resistant to imatinib (Figure 1B). These secondary mutations are invariably found in *cis* with the primary mutation, but patients with primary resistance, progression within the first six months of treatment, to imatinib generally do not show secondary mutations. However, most patients who show secondary resistance (defined as progression after the first six months of treatment, typically within 2 years of treatment) will show secondary mutations. Primary mutations in exon 11 are more sensitive to TKI than those with exon 9 primary mutations; however, secondary resistance mutations are more frequent in patients with primary exon 11 mutations than in patients with primary exon 9 mutations. In terms of the effect of secondary mutations on the benefits of TKI treatment, patients with the KIT ATP-binding domain mutations (V654A and T670I) or with the activation loop mutations in exons 17 and 18 do worse on imatinib (Figure 1B). However, sunitinib effectively overcomes the ATP-binding pocket mutants, but not the activation loop mutants. Other drugs are proving to exert specific effects on different mutations in clinical trials. For example, sorafenib is showing remarkable clinical effects against the gatekeeper mutation T670I [79, 80]. Imatinib resistance was also identified in GISTs with PDGFRA mutations, and the major mutation is D842V mutation [74, 81]. Viable tumor cells are found in most patients who have had their tumors resected during imatinib therapy, suggesting that a certain level of drug resistance is inherent in most GISTs. Many secondary KIT mutations have been found at low frequencies, and mutations often exhibit inter-tumor or even intra-tumor heterogeneity in the same patient [69, 76, 82, 83]. This heterogeneity is unfavorable for the subsequent treatment of imatinib resistance.

### Strategy to overcome resistance

Several approaches are available to combat imatinib resistance (Table 2). First, increasing the dose of imatinib is recommended, but the effect of this strategy is unfortunately limited. Presumably, imatinib resistance is caused by either inadequate drug concentrations, associated secondary KIT mutations or activation of signaling pathways that by pass KIT, and higher drug concentrations are thought to inhibit tumor growth in relative rather than absolute imatinib resistance [84]. In addition to the many new TKIs, including sunitinib and regorafenib, that are being developed, and other inhibitors that target GIST via mechanisms independent of *KIT* or *PDGFRA* are being developed [85–87].

**Table 2:** Novel therapies being tested for the treatment of GISTs

Drug	Key targets	Manufacturer	References
***Tyrosine kinase inhibitors***			
BLU-185	KIT, PDFRA	Blueprint Medicines	[87]
Dasatinib	KIT, PDGFRs, BCR-ABL, SRC	Bristol-Myers Squibb	[88]
Pazopanib	KIT, PDGFRA, VEGFR1-3	GlaxoSmithKline	[89]
Masitinib	KIT, PDGFR, FGFR3, Lyn, FAK	AB science	[90]
***Monoclonal antibodies***			
LOP628	KIT	Novartis	[91]
Ipilimumab	CTLA4	Bristol-Myers Squibb	[92]
***Signaling pathway inhibitors***			
Binimetinib	MEK	Novartis	[93]
Alpelisib	PI3K	Novartis	[94]
***Others***			
Palbociclib	CDK4/6	Pfizer	[95]
Onalespib	HSP90	Astex Pharmaceuticals	[97]
Flavopiridol	Transcription of *KIT*	Tolero Pharmaceuticals	[77]

#### Tyrosine kinase inhibitors

**BLU-285** (Blueprint Medicines, Cambridge, MA, USA) is an orally available agent that was developed to specifically target the mutant forms of *KIT* and *PDGFRA*. It potently and selectively inhibits exon 17-mutated KIT and the *PDGFRA* D842V mutant [88].

**Dasatinib** (Sprycel, Bristol-Myers Squibb, New York, NY, USA) is a BCR-ABL inhibitor approved as the first-line drug in patients with CML. The main targets of dasatinib are BCR-ABL, SRC, KIT, PDGFRs and several other tyrosine kinases. Combination with dasatinib plus imatinib or ipililumab has better efficiency for blockade of KIT signaling [89].

**Pazopanib** (Votrient, GlaxoSmithKline, Brentford, UK) is a broad-spectrum tyrosine kinase inhibitor that is efficacious against KIT, FGFR, PDGFR and VEGFR, as reported at the 2015 Annual Meeting of ASCO [90]

**Masitinib** (Masivet, AB Science, Paris, France) is an orally available TKI with more efficiency than imatinib against *KIT* exon 11-mutated and wild-type in GIST. It also effectively inhibits PDGFR and FGFR. The compound has already undergone several clinical trials to study its efficacy and safety and compare them to those of sunitinib. This study showed that masitinib prolonged survival and had a better safety profile than sunitinib, although the progression-free survival curves for these two drugs were similar. GIST patients with no evidence of disease after surgery but with a high potential of recurrence will be given with masitinib, and a phase 3 study is being planned to test masitinib in the adjuvant setting and evaluate its efficacy and safety in the first-line setting for GIST [91].

#### Monoclonal antibodies

**LOP628** (Novartis, Cambridge, MA, USA) is a humanized monoclonal antibody targeting the KIT receptor and is linked to the cytotoxic agent maytansine, which inhibits the assembly of microtubules, molecules that are necessary for cell division. The mechanism of action of this “antibody-drug conjugate” requires two steps. First, the monoclonal antibody portion of LOP628 targets and binds to the cell surface receptor KIT that is expressed on all GIST cells. This process is followed by the internalization of the LOP628-KIT complex and binding of the maytansine portion of LOP628 to the tubulin molecules inside the cell, which inhibits cell division and tumor growth. The FDA has approved a similar concept to treat advanced breast cancer with HER2(+) using an anti-HER2 antibody (trastuzumab, Herceptin) conjugated to the cytotoxic maytansinoid DM1 (trastuzumab emtansine). This therapeutic concept has great potential, especially for the treatment of GIST, because most GISTs depend on the activation of KIT signaling, even when they are resistant to TKIs. LOP628 can only bind to KIT-expressing cells, but its activity is not inhibited by secondary resistance mutations.

Another monoclonal antibody is ipilimumab (BMS, New York, NY, USA) in combination with dasatinib. Ipilimumab binds cytotoxic T-lymphocyte-associated antigen-4 (CTLA4) directly, a protein receptor enriched on T cells that functions as an immune checkpoint, to downregulate the immune system. The inhibition of CTLA4 can re-activate the known-tumor activities of cytotoxic T cells, and augmenting the immune system with a CTLA4 inhibitor significantly enhanced the effect of imatinib treatment [92].

#### Signaling pathway inhibitors

Several trials are testing inhibitors to block downstream pathways of KIT in conjunction with imatinib. One prospective study is using the MEK inhibitor MEK162 (Binimetinib, Novartis, Cambridge, MA, USA), combining imatinib in advanced GISTs. This strategy is based on the discovery that MEK/MAPK signaling activates ETV1, a lineage-specific survival factor for GIST and its precursor, interstitial cells of Cajal. In preclinical studies, MEK162 synergistically destabilized ETV1 protein with imatinib and suppressed GIST formation and progression [93]. Another inhibitor, BYL-719 (Alpelisib, Novartis, Cambridge, MA, USA), is a selective inhibitor of the PI3K catalytic p110α subunit. Notably, neither compound significantly inhibited the downstream kinase mTOR, which is known to provide a negative feedback loop that reactivates the PI3K and the MAPK pathways [94].

#### Others

The efficacy and safety of Palbociclib (Ibrance, Pfizer, New York, NY, USA) for advanced GISTs who are refractory to imatinib and sunitinib are also being evaluated. PD-0332991 is an oral inhibitor of the CDK4 and CDK6, which are crucial promoters of cell division and often deregulated in cancer, including GIST. In fact, a publication by Bauer et al. showed that defects in the cell cycle are the most common aberrations in GIST after *KIT/PDGFRA* mutations.

**AT13387** (Onalespib, Astex Pharmaceuticals, Pleasanton, CA, USA) is a *KIT* chaperone HSP90 inhibitor [23, 95], which has been shown to be effective against imatinib-resistant GIST [96].

**Flavopiridol** (Alvocidib, Tolero Pharmaceuticals, Lehi, UT, USA) can repress the transcription of *KIT* to downregulate the activity of pathways downstream of KIT.

The genotype of GISTs is very relevant to the response of the tumor to various drugs. Thus, more research is needed to identify mutations that endow resistance to a particular treatment and effective therapies for each unique genotype. As previously mentioned, imatinib-resistant tumors are highly heterogeneous. Thus, the use multiple agents to confront different secondary mutations is reasonable [97, 98]. Theoretically, a cocktail of inhibitors could suppress tumor growth by inhibiting multiple pathways. However, combining small-molecule drugs for synchronous treatment may be a challenge because most inhibitors can be metabolized through cytochrome P450 signaling pathways [99]. Therefore, the composition and effects of multiple agents should be explored further.

## CONCLUSIONS

**Figure 4:**
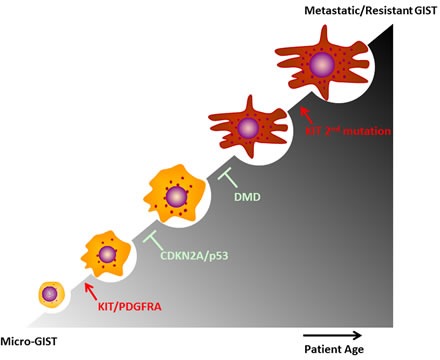
Major genomic alterations promote GIST progression

GISTs are common neoplasms, and so-called micro GISTs are found in ∼20-30% of adults. Gain-of-function *KIT* or *PDGFRA* mutations are early genetic events (Figure 4), and most GISTs exhibit remarkable clinical responses to TKIs, including imatinib, sunitinib and regorafenib. The clinical success of TKIs in GIST serves as a paradigm for the molecularly targeted therapy of solid tumors. Unfortunately, even patients with dramatic initial clinical responses ultimately develop resistance to TKI therapy, resulting in disease progression. Genomic alterations contribute to tumorigenic progression in GIST, including cell cycle abnormalities due to CDKN2A or TP53 loss-of-function, dystrophin inactivation, 1p, 14q, 22q alterations and secondary KIT mutations (Figure 4). Further genomic screens pave the way to identify more drivers in GIST tumorgenesis that have a potential to be validated as a target, and novel insights into the genetic progression of GISTs are shedding new light on therapeutic innovations.

## Abbreviations

Aos: Antisense oligonucleotides
CDK4 and CDK6: Cyclin dependent kinase 4 and 6
CDKN2A: Cyclin-dependent kinase inhibitor 2A
CML: Chronic myelogenous leukemia
CSF1R: Colony-stimulating-factor receptor
CTLA4: Cytotoxic T-lymphocyte-associated antigen-4
ER: Estrogen receptor
FDA: Food and Drug Administration
FGFR: Fibroblast growth factor receptor
FLT3: Fms-like tyrosine kinase 3
Fzd7: Frizzled-7
GISTs: Gastrointestinal stromal tumors
HER2: Human epidermal growth factor receptor 2
HR: Hazard ratio
HSP90: Heat shock protein 90
ICC: Interstitial cells of Cajal
JNK: c-Jun N-terminal kinase
LMS: Leiomyosarcoma
LOH: Loss of heterozygosity
NF-κB: Nuclear factor kappa-light- chain-enhancer of activated B cells
PDGFRA and PDFGRB: Platelet-derived growth factor receptor α and β
PDN: Prednisolone
PR: Partial response
Rb: Retinoblastoma
RET: Rearranged during transfection
RMS: Rhabdomyosarcoma
SCF: Stem cell factor
SD: Stabilized disease
STAT3: Signal transducer and activator of transcription 3
TKI: Tyrosine kinase inhibitor
TNF: Tumor necrosis factor
VEGFR: Vascular endothelial growth factor receptor
